# Pharmacokinetically guided phase I trial of topotecan and etoposide phosphate in recurrent ovarian cancer

**DOI:** 10.1038/sj.bjc.6602657

**Published:** 2005-06-14

**Authors:** N C Levitt, D J Propper, S Madhusudan, J P Braybrooke, C Echeta, R te Poele, S L Davies, E Flanagan, I D Hickson, S Joel, T S Ganesan

**Affiliations:** 1 Cancer Research UK Cancer Centre, Churchill Hospital, Oxford OX3 7LJ, UK; 2 Cancer Research UK, Medical Oncology Unit, St Bartholomew's Hospital, West Smithfield, London EC1A 7BE, UK; 3 Genome Integrity Group, Molecular Oncology Laboratories, Weatherall Institute of Molecular Medicine, John Radcliffe, Hospital, Oxford 0X3 9DS, UK; 4 Bristol Haematology and Oncology Centre, Horfield Road, Bristol BS2 8ED, UK

**Keywords:** topotecan, etoposide phosphate, topoisomerases, ovarian cancer

## Abstract

A pharmacokinetically guided phase I study of topotecan and etoposide phosphate was conducted in recurrent ovarian cancer. The scheduling of the topoisomerase I and II inhibitors was determined using *in vitro* activity data. All patients had recurrent disease following prior platinum-containing chemotherapy. Patients had a World Health Organisation performance status of 0–2 and adequate bone marrow, renal and hepatic function. Treatment was with topotecan intravenously for 5 days followed immediately by a 5-day intravenous infusion of etoposide phosphate (EP), with pharmacokinetically guided dose adjustment. Plasma etoposide levels were measured on days 2 and 4 of the infusion. A total of 21 patients entered the study. In all, 48% were platinum resistant and 71% had received prior paclitaxel. The main toxicities were haematological, short lived and reversible. A total of 29% of patients experienced grade 4 thrombocytopenia and 66% grade 4 neutropenia after the first cycle. Neutropenia and thrombocytopenia was dose limiting. The maximum-tolerated dose was topotecan 0.85 mg m^−2^ day^−1^ days 1–5 followed immediately by a 5-day infusion of EP at a plasma concentration of 1 *μ*g ml^−1^. The response rate (RR) was 28% in 18 evaluable patients. There was marked interpatient variability in topoisomerase II*α* levels measured from peripheral lymphocytes, with no observed increase following topotecan. This regimen of topotecan followed by EP demonstrated good activity in recurrent ovarian cancer and was noncrossresistant with paclitaxel. Both the toxicity and RR was higher than would be expected from the single agent data, in keeping with synergy of action.

Advanced ovarian carcinoma is usually sensitive to chemotherapy with durable complete remissions but relapse often occurs, necessitating further treatment. Evaluation of appropriate active regimens for patients with recurrent or resistant ovarian cancer allows the possibility of eventually incorporating them into initial management (Stuart, 2003). The initial treatment for advanced ovarian cancer comprises cytoreductive surgery followed by platinum-containing chemotherapy with or without paclitaxel (McGuire *et al*, 1996; Muggia *et al*, 2000; Piccart *et al*, 2000; ICON3 collaborators, 2002). Effective noncrossresistant second- and third-line therapies are required. This study evaluates the sequential combination of topotecan and etoposide phosphate (EP).

Topotecan (Hycamtin; SmithKline Beecham Pharmaceuticals, Philadelphia, PA, USA) is a semisynthetic analogue of the plant alkaloid camptothecin which is a topoisomerase I inhibitor and is active in ovarian cancer. According to phase I trials, the best schedule and maximum-tolerated dose (MTD) of topotecan is 1.5 mg m^−2^ day^−1^ for 5 consecutive days in a 21-day cycle (Rowinsky and Verweij, 1997). In phase I studies of topotecan, the main dose-limiting side effect was neutropenia, which was predictable, of short duration and noncumulative. Other side effects included thrombocytopenia, alopecia and gastrointestinal toxicity. Mucositis, fatigue, rashes and abnormalities of liver function were also rarely reported. Phase II studies have shown activity and prolonged disease stabilisation in patients with recurrent ovarian cancer (Creemers *et al*, 1996; Kudelka *et al*, 1996). This was also observed in patients whose disease had progressed following prior chemotherapy with paclitaxel or platinum agents (Bookman *et al*, 1998; Gore *et al*, 2001). A randomised phase III study that compared topotecan to paclitaxel as second-line therapy after platinum-containing regimens showed a higher response rate (20.5 *vs* 13.2%, *P*=0.138), longer response duration (32 *vs* 20 weeks, *P*=0.222) and longer time to progression (23 *vs* 14 weeks, *P*=0.002) for topotecan, although there was no significant improvement in survival (ten Bokkel Huinink *et al*, 1997). In view of these studies, topotecan has been licensed as a second-line therapeutic agent for ovarian cancer. More recently, a shorter 3-day infusion of topotecan 1.5 mg m^−2^ day^−1^ in advanced ovarian cancer has shown similar activity but was less toxic than standard treatment (Markman *et al*, 2000).

Etoposide, a topoisomerase II inhibitor, has antitumour activity in a wide range of tumours including small-cell lung cancer (SCLC), germ cell tumours of the testis and lymphomas. Etoposide has been given in a variety of schedules both as a single agent and in combination therapy in a number of tumour types (Hande, 1998). Response to etoposide is dependent on drug scheduling as its activity is specific to S-phase of the cell cycle (Joel *et al*, 1998).

Oral etoposide has been investigated as a route of prolonged administration (Hainsworth *et al*, 1989). Although oral schedules maintain low plasma etoposide levels throughout the treatment period, plasma concentrations still vary between peak and pretreatment levels. Additionally, the absorption of oral etoposide is incomplete (60% bioavailability) and variable, both between and within patients (Harvey *et al*, 1985b; Slevin and Joel, 1993). Phase II studies with oral etoposide showed response rates of 24 and 26% in patients with relapsed or platinum-resistant ovarian cancer (Hoskins and Swenerton, 1994; Seymour *et al*, 1994). Myelotoxicity was dose limiting in both studies. A third phase II study showed response rates of 34.1% in platinum-sensitive patients and 26.8% in platinum-resistant patients. Almost one-third of platinum-resistant responders had also received paclitaxel (Rose *et al*, 1998). The main toxicities were haematological. Thus, oral etoposide is noncrossresistant with both platinum agents and paclitaxel.

EP (Etopophos, Bristol Myers Squibb Co, Princeton, NJ, USA) is rapidly converted to etoposide in the blood. It is approximately 20 times more soluble than etoposide and can therefore be given as a prolonged outpatient infusion. A recent phase II study has evaluated the efficacy of EP in patients with relapsed or resistant ovarian carcinoma, previously treated with both carboplatin and paclitaxel (O'Byrne *et al*, 1997). To ensure that target concentrations were achieved rapidly, a loading dose of EP was given intravenously over 30 min. At the same time, a constant infusion of EP was commenced to maintain plateau levels at the target concentration. Plasma drug monitoring was performed 24 h into the infusion and at 3 days, with infusion rate modification if necessary to ensure that target levels were achieved. In the first cohort of 16 patients treated with EP to achieve a plasma level of 2 *μ*g ml^−1^, no objective responses were observed although disease stabilisation occurred in four patients. The next cohort of patients were treated at 3 *μ*g ml^−1^. Of 28 patients, 26 were evaluable and the response rates were 43% for platinum-sensitive patients and 17% for platinum-resistant patients. In all, 20% of patients in the second cohort experienced grade 4 myelosuppression, suggesting that in heavily pretreated patients with ovarian cancer, the single agent dose of EP is 3 *μ*g ml^−1^ infused for 5 days.

There are theoretical reasons and some preclinical data to suggest that topotecan and etoposide may act synergistically in sequential combination with the schedule of administration being important (Whitacre *et al*, 1997). A proposed mechanism for synergy is that topoisomerase I inhibitors increase topoisomerase II*α* levels, sensitising malignant cells to the effects of topoisomerase II inhibition (Whitacre *et al*, 1997). Thus, the sequential administration of topotecan followed by EP might lead to total topoisomerase ‘shutdown’ and synergistic antitumour activity.

This study assesses the combination of a 5-day schedule of topotecan followed closely by a continuous 5-day infusion of EP for toxicity and to identify the MTD of both drugs, in patients with recurrent or platinum-resistant ovarian carcinoma. In addition, the combination of both drugs was studied *in vitro* to evaluate the schedule and potential synergy.

## PATIENTS AND METHODS

### Eligibility

The study was open to patients with histologically confirmed epithelial ovarian carcinoma, primary serous papillary peritoneal carcinoma or fallopian tube carcinoma and evaluable, relapsed or platinum-resistant disease. Platinum-resistant disease was defined as progression on treatment or relapse within 6 months of completion of initial treatment. Patients were required to be over 18 years old and with a World Health Organisation (WHO) performance status of 0–2. They were required to have adequate bone marrow (neutrophils ⩾1.5 × 10^9^ l^−1^, platelets ⩾100 × 10^9^ l^−1^), renal (normal serum creatinine and EDTA clearance >40 ml min^−1^) and hepatic function (serum bilirubin <50 *μ*mol l^−1^, albumin >30 g l^−1^, aspartate amino transferase and alkaline phosphatase ⩽3 times the upper limit of normal or ⩽5 times the upper limit of normal in the presence of liver metastases). All patients had recovered from the acute toxic effects of previous treatment and had not received radiotherapy, chemotherapy or immunotherapy within 4 weeks of trial entry. Pregnant women, nursing mothers and patients not using adequate contraception were excluded, as were patients with clinical evidence of cerebral metastases. All patients gave written informed consent for the trial and for insertion of a Hickman line to allow drug delivery. The trial was approved by the local research ethics committee and was conducted according to the recommendations of the Declaration of Helsinki, Hong Kong Amendment 1989 and the ABPI guidelines for good clinical practice.

### Study design

This single centre phase I study used a modified Fibonacci design with a planned minimum of three patients at each dose level. As both chemotherapy agents in this study are myelotoxic, topotecan was given at 1.0 mg m^−2^ day^−1^ consecutively for days 1–5, two-third of its usual recommended dose. This was followed by a continuous infusion of EP, achieving a plasma concentration of etoposide of 2 *μ*g ml^−1^ for days 6–10 in dose level 1a and adjusted thereafter according to toxicity. Dose levels used in this study are listed in Table 1. This starting dose was chosen following a phase II study of EP alone (O'Byrne *et al*, 1997), in which a dose level of 2 *μ*g ml^−1^ for 5 days was well tolerated. Patients were retreated in four weekly cycles. Standard antiemetic cover was with metoclopramide, as required. Dexamethasone and tropisetron were used if patients had persistent nausea. Patients were also routinely given warfarin 1 mg once daily as prophylaxis against Hickman line-induced venous thrombosis. Granulocyte colony-stimulating factor was not given in this study.

Dose escalation to higher levels was planned providing that no patient in the cohort experienced dose-limiting toxicity (DLT). If however, two out of three or three out of six patients in a single dose level experienced DLT, then it was planned to stop recruiting at that level and to include additional patients at a lower dose level. As it was anticipated that myelotoxicity might be dose limiting even at dose level 1a, further (lower) dose escalation schemes were devised. As patients who have received both paclitaxel and carboplatin in the past may have less marrow reserve than patients who have only received carboplatin, it was planned to expand the MTD level to include at least four patients who had previously been treated with both agents.

Dose-limiting toxicity was defined using the CALBG (Cancer and Leukemia Group B) common toxicity grading, as grade 4 haematological toxicity during the first cycle of treatment (grade 4 thrombocytopenia or grade 4 neutropenia lasting for more than 5 days or complicated by fever), or grade 3 nonhaematological toxicity (excluding nausea, vomiting and alopecia) or grade 4 vomiting in patients with maximal antiemetic supportive care. As it was probable that myelotoxicity would be cumulative, only first cycle effects were defined as contributing to DLT. The stop dose was defined as the dose at which two out of three or three out of six patients experienced DLT. Maximum-tolerated dose was defined as the highest dose level at which no more than one out of six patients experienced DLT. The MTD is therefore the dose below the stop dose level.

In the 2 weeks prior to recruitment into the study, patients were assessed by clinical examination and with a chest radiograph, EDTA (ethylene diamine tetra-acetic acid) clearance, full blood count and differential, serum electrolytes, including calcium and phosphate, creatinine and urea, liver function tests, Ca125 and urinalysis. Within 4 weeks of recruitment, all patients had full assessment of any measurable/evaluable lesions by CT scan of abdomen and pelvis and by other investigations such as clinical photography, CT thorax, MRI or bone scans as clinically indicated. Blood tests and urinalysis were repeated on the day prior to starting treatment, before each subsequent cycle and 2 weeks after completing the study. Dose reductions to the next lowest dose level were made if patients experienced any grade 4 haematological or grade 3 nonhaematological toxicity (excluding alopecia). Treatment in subsequent cycles was delayed until the toxicity had resolved. Quality of life (QOL) was assessed with the EORTC (European Organisation for Research and Treatment of Cancer) 30 questionnaire prior to cycles 1, 3 and 6.

Patients, who tolerated the chemotherapy and had stable or responding disease after three cycles, were continued on study for a maximum of six cycles. If there was evidence of progressive disease, then treatment was stopped after a minimum of two cycles. The response to treatment was formally assessed after three and six cycles. Disease response was assessed according to World Health Organisation (WHO) criteria. The duration of response was calculated as the period from the final course of chemotherapy through to the date of confirmed relapse.

### Drug formulation and administration

Topotecan was reconstituted in sterile saline and given as an infusion via a Hickman line over 30 min, on days 1–5 of each cycle. Bristol-Myers Squibb Pharmaceuticals supplied EP, as 113.6 mg of lyophilised powder in a sterile glass vial. Each vial was reconstituted with sterile saline. Starting on day 6, EP was infused via a Hickman line, starting with a loading dose of 10 × target etoposide level mg m^−2^, given over 30 min, followed by a 5-day continuous infusion in mg m^−2^ h^−1^. The initial infusion rate was calculated and adjusted according to measured plasma etoposide concentrations (Joel *et al*, 1996).

### Therapeutic drug monitoring

Etopside phosphate was commenced on the afternoon of day 6. On the morning of day 7 (18 h after the start of the infusion) and on day 9 of each cycle, peripheral venous blood samples were drawn for determination of total plasma etoposide levels, as described previously (Harvey *et al*, 1985a; Joel *et al*, 1996, 1998). Plasma standards covering the range 0.5–5.0 mg ml^−1^ were used; patient samples were run in duplicate, with quality control samples at two concentrations (1.25 and 3.5 mg ml^−1^). Between-assay reproducibility based on these quality control samples was <10%. Plasma etoposide concentration was obtained on the day of sampling, with a typical turn-around time of 3–4 h to permit adjustment of the infusion rate on that day. Dose modifications were made according to the measured steady-state plasma etoposide concentration using the following formula: (Joel *et al*, 1996) 
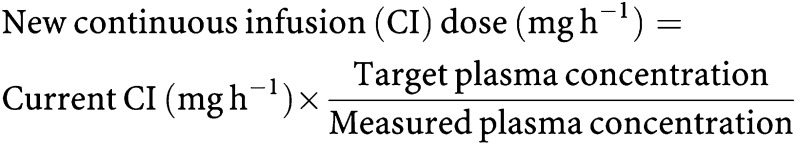


Adjustments to the infusion rate were only made if the measured plasma etoposide concentration differed by >10% from the target concentration. The initial infusion rates for cycles 2–6 were based on the plasma etoposide concentration from the preceding cycle.

### Preclinical evaluation of cell lines

Ovarian cancer cell lines (A2780, SKOV-3 and OVCAR-3) were cultured in RPMI 1640 medium supplemented with 10% foetal bovine serum, penicillin 100 U ml^−1^ and streptomycin 100 *μ*g ml^−1^ (Gibco BRL, Paisley, UK). Cells were incubated at 37°C in a humidified atmosphere containing 5% CO_2_. Stock solutions of the topoisomerase I inhibitor, SN-38 (gift from Aventis Pharma, France) were prepared in DMSO (dimethylsulphoxide, Sigma, Dorset, UK), and etoposide (Sigma) in 50% methanol. These were diluted to the appropriate concentrations in media immediately prior to use. The final concentration of DMSO in the culture medium was <0.02%. Cells were plated at a density of 2 × 10^5^ ml^−1^ and allowed to attach for 24 h prior to drug treatment. The activity of SN-38 and etoposide as single agents was first determined across a range of concentrations (0–100 ng ml^−1^ SN-38, 0–10 *μ*M etoposide). The concentration of each drug resulting in 10% apoptosis after 3 days was determined from concentration effect curves using a sigmoidal *E*_MAX_ model and used in subsequent combination experiments. These concentrations were 3.125 ng ml^−1^ for SN-38 in each cell line, 2 *μ*M etoposide in A2780, 1 *μ*M etoposide in OVCAR-3 and 3 *μ*M etoposide in SKOV3. Drug medium was replaced every 24 h, with detached cells harvested by centrifugation and resuspended in the fresh drug medium.

Cell cycle distribution, including the apoptotic (sub-G0/G1) population, was determined using propidium iodide following the controlled extraction of low molecular weight DNA. Cells were harvested by trypsinisation and centrifugation, and fixed in 70% ethanol overnight. After removal of fixative, cells were resuspended in 750 *μ*l of DNA extraction buffer (96% 0.2 M Na_2_HPO_4_, 4% 0.1 M citric acid) for 5 min at room temperature. Buffer was removed by centrifugation and the cells were resuspended in DNA staining solution (50 *μ*g ml^−1^ propidium iodide and 50 *μ*g ml^−1^ ribonuclease A, Sigma) for 1 h in the dark. Fluorescence data from 10 000 cells was collected on a FACScan flow cytometer (Becton Dickinson) and analysed using LYSYS II software. Results presented are the mean of a minimum of three separate experiments. Statistical comparisons were made using a paired *t*-test.

### Peripheral blood topoisomerase II*α* levels

In all, 10 ml blood samples were taken into iced cold EDTA containers on days 1, 6 and 10 of cycle one for topoisomerase II*α* analysis. Samples were processed immediately; 5 ml of blood was layered onto 5 ml lymphoprep (Nycomed Pharma AS Diagnostics, Oslo, Norway) and centrifuged for 20 min at 4°C. The lymphocyte layer was washed once with phosphate-buffered saline and recentrifuged. The cell pellet was resuspended in 2 × SDS sample buffer and incubated at 90°C for 10 min before freezing at −70°C. Polyacrylamide gel electrophoresis and Western blotting was performed as previously described (Ausubel *et al*, 1994). Blots were probed with a monoclonal anti-topoisomerase II*α* antibody, 1F6 (Negri *et al*, 1992). *β*-Tubulin (monoclonal anti-*β*-tubulin clone 2.1, Sigma, Missouri, USA) was used as a loading control. Autoradiographs were scanned and the band intensity of the lanes compared using FluorChem software, with the tubulin band as a control for each sample.

## RESULTS

### Patients and treatment

A total of 21 patients were entered into this study (Table 2). One patient's performance status fell from 2 when she was consented for the trial to 3 on day 1 of the study. All patients had received previous platinum-containing chemotherapy and 15 out of 21 (71%) had also received paclitaxel (Table 3a). Only two patients (10%) progressed through first-line platinum-containing chemotherapy. A total of 10 patients (48%) either progressed following first-line chemotherapy or had duration of response of less than 6 months and were therefore classified as platinum-resistant (Table 3a). A total of 15 patients had received two or more chemotherapy regimens (Table 3b). One patient had had prior exposure to etoposide, given orally as third-line chemotherapy.

In this study, a total of 100 cycles of trial chemotherapy were given with a mean of 4.8 cycles per patient. A total of 12 patients completed all six planned cycles of chemotherapy.

### Toxicity

The main toxicity was myelosuppression (Table 4). Both patients entered at dose level 1a (T-1.0/E-2.0) experienced grade 4 neutropenia with associated sepsis following the first cycle of treatment and therefore the next patient was recruited to the lower dose level of 1b (T-0.85/E-2.0). This dose level also proved to be excessively myelotoxic, with all three patients suffering grade 4 neutropenia, two with associated sepsis. Dose level 2a (T-0.85/E-1.0) was better tolerated and was therefore expanded to 11 patients in order to allow for better assessment of toxicity at this dose level. Four of these patients experienced grade 4 neutropenia, however this was for fewer than 5 days and was complicated by sepsis in only one case. As DLT at dose level 2a was observed in only one of 11 patients, a higher dose level, 2b (T-0.85/E-1.5) was given to a further five patients. Dose-limiting toxicity was also seen at this dose level, with grade 4 neutropenia complicated by sepsis in four out of five patients. Of 13 patients with grade 4 neutropenia during cycle 1, 10 became septic. This high incidence of neutropenic sepsis may reflect the heavy pretreatment of this patient group and the fact that neither prophylactic antibiotics nor G-CSF were given in this trial. There were no deaths from neutropenic sepsis. Of note, the haematological toxicity in this study was of short duration with only three out of 21 patients having a treatment delay of ⩾1 week at cycle 2. Nonhaematological toxicity was not dose limiting, detailed in Table 4. Generally this was mild and manageable with grade 3 toxicities of nausea, stomatitis and diarrhoea seen in three, one and one patients, respectively.

### Pharmacokinetics

**Figure 1:**
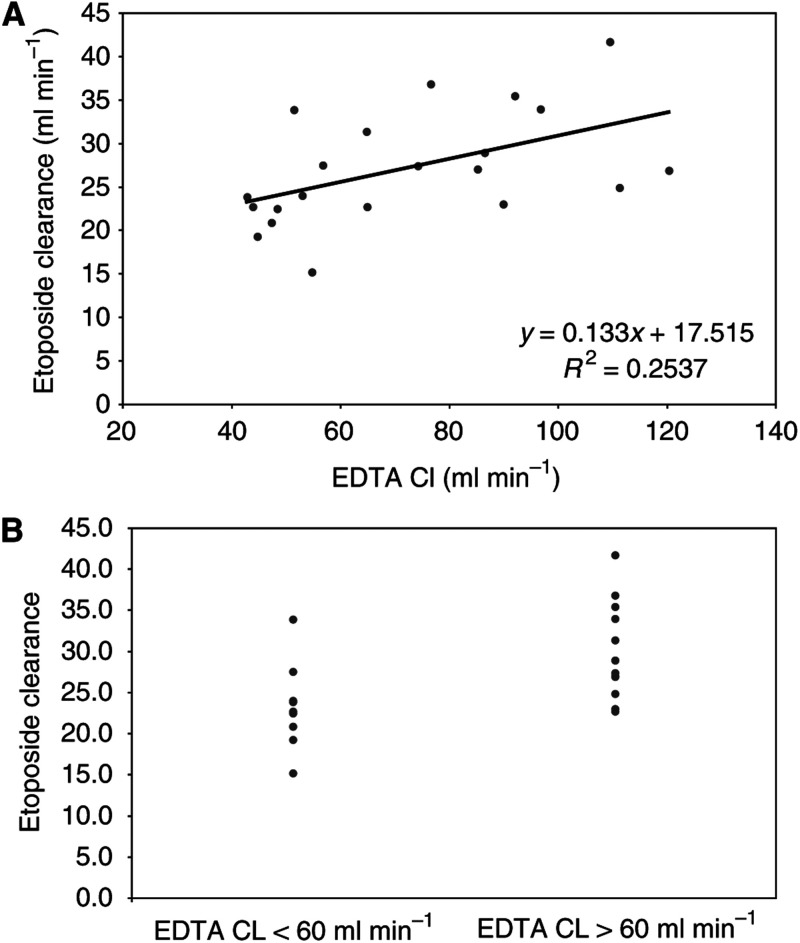
Etoposide clearance. Relationship between etoposide clearance and EDTA clearance (renal function) prior to treatment, shown as a regression curve (**A**) and as a scatter graph for EDTA clearance below and above 60 ml min^−1^ (**B**).

All patients had pharmacokinetic sampling during each treatment cycle, with adjustment of the infusion rate to achieve the targeted plasma etoposide level. The starting infusion rate for cycle 1 did not allow for differences in renal function, as the total etoposide plasma clearance correlated only poorly with the EDTA creatinine clearance (Figure 1). The starting infusion rate for cycle 2 and subsequent cycles was the same as the final infusion rate from the preceding cycle.

**Figure 2:**
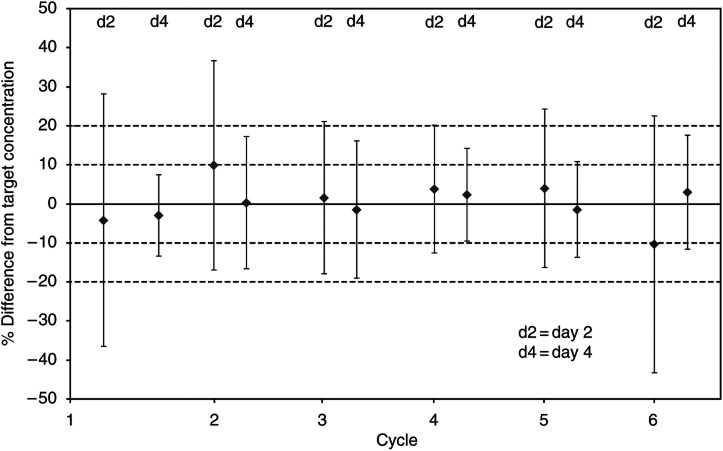
Difference from target concentration. Graph demonstrating the variability of plasma etoposide concentrations, measured on days 2 and 4 of the infusion, for each cycle.

There was considerable interpatient variability in the measured plasma etoposide levels (Figure 2). Across all cycles, samples from day 2 showed that 32% of patients had measured plasma etoposide levels within 10% of the target level. This improved to a mean of 58% for day 4 measurements. Measured etoposide levels were within 20% of the target level for 66% of patients on day 1 and 85% by day 4. Patients with measured etoposide levels more or less than 10% from the target level had their etoposide phosphate infusion adjusted. Table 5 details the number of patients for each cycle for whom this was necessary.

### Tumour responses at the end of treatment

A total of 18 patients were evaluable for tumour response, having completed at least three cycles of treatment. Objective tumour responses were demonstrated in 28% (five out of 18) of patients at completion of treatment (Table 6). All responding patients had been sensitive to platinum first line and four out of five had also been treated previously with paclitaxel. All of the 18 evaluable patients had an elevated Ca125 level at the start of the trial. Six patients had a ⩾50% fall in their level, and in a further five patients the Ca125 levels fell below baseline but by less than 50% after six cycles. Of the 21 patients, 11 went onto between 1 and 3 (mean 1.5) further regimens of chemotherapy, after completion of chemotherapy in this study. The median survival for patients was 11.7 months from the start of treatment on this study, with all patients having died at the time of this report.

### Quality of life

**Figure 3:**
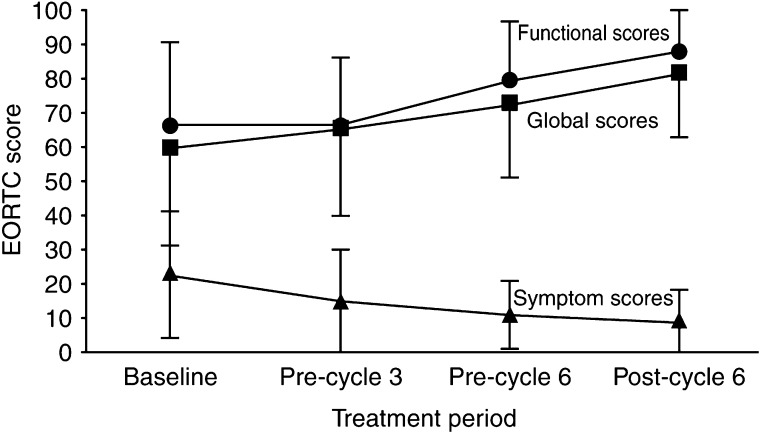
European Organisation for Research and Treatment of Cancer (EORTC) score. Mean EORTC QOL scores (±1 s.d.) calculated at baseline, before cycles 3 and 6 and after cycle 6.

Quality of life was measured during the treatment using the EORTC QL-30 questionnaire (Figure 3). Quality of life was statistically related to patients' haemoglobin level and to their level of symptoms. There was a trend for QOL to improve during the trial; however, the difference was not statistically significant.

### Preclinical studies

**Figure 4:**
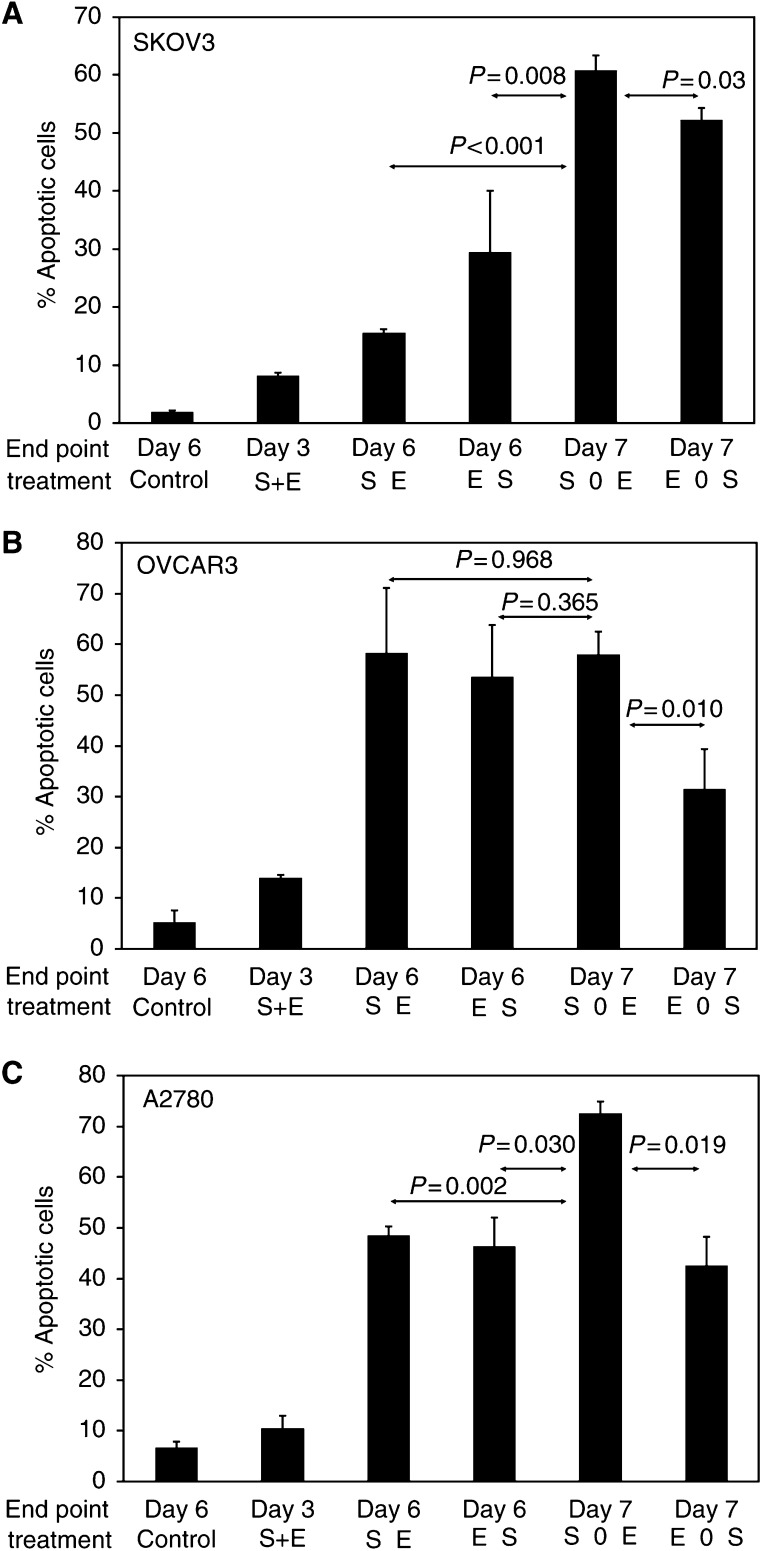
Percentage of apoptotic cells. Apoptosis (mean+s.d.) induced by combinations of etoposide and SN-38 in SKOV-3 (**A**), OVCAR-3 (**B**) and A2780 (**C**) ovarian cancer cell lines. The treatments were; Control, SN-38+etoposide concurrently for 3 days (S+E), SN-38 for days 1–3 immediately followed by etoposide for days 3–6 (SE), etoposide for days 1–3 immediately followed by SN-38 for days 3–6 (ES), SN-38 for days 1–3 followed by 1 day drug free, then etoposide for days 4–7 (S0E), etoposide for days 1–3 followed by 1 day drug free, then SN-38 for days 4–7 (E0S).

Apoptosis was assessed following exposure to etoposide and SN-38 in combination (Figure 4A–C). With the exception of SN-38 in A2780 cells, where the response was mainly cell cycle arrest even up to 100 ng ml^−1^, SN-38 drug concentrations used were those that resulted in approximately 10% apoptosis after a 3-day exposure. The percentage of apoptotic cells in each experiment was determined by flow cytometry using propidium iodide staining of DNA on the last day of each treatment.

For each cell line, exposure to both drugs simultaneously resulted in less apoptosis than would be expected from the additive effect of each drug given separately. Schedules in which the agents were used consecutively were consistently more active than those involving concurrent exposure, even though the overall exposure (concentration × time) for both agents was the same in each schedule. The most active combination overall, as evaluated by induction of apoptosis, was SN-38 for 3 days, followed by 1 day drug free and then etoposide for a further 3 days in all cell lines (Figure 4A–C).

### Topoisomerase II*α* levels

**Figure 5:**
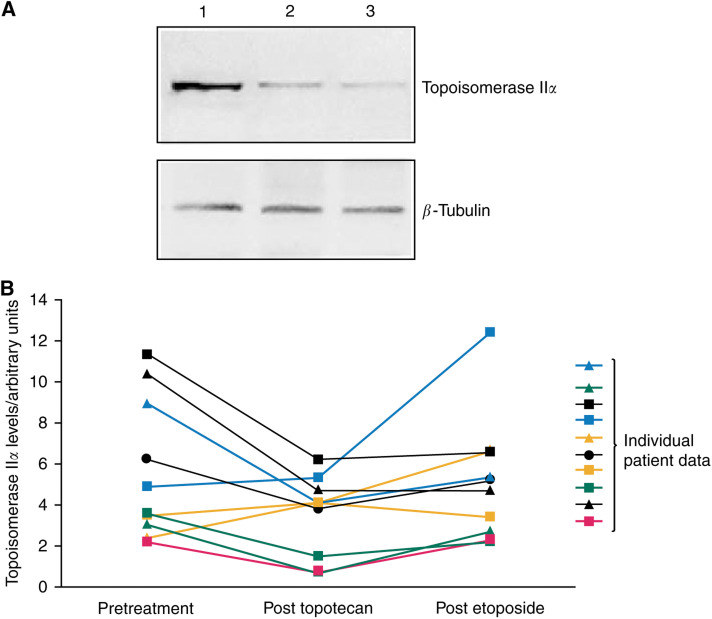
Topoisomerase II*α* levels/arbitrary units. Topoisomerase II*α* levels in peripheral blood. An example Western blot of topoisomerase II*α* (**A**). Lymphopreps were made from peripheral blood samples taken pretreatment (1), post-topotecan (2) and post-etoposide (3) administration. Western blotting was performed on proteins from the lymphocyte cell pellet. Blots were probed with a monoclonal anti-topoisomerase II*α* antibody. *β* Tubulin was used as a loading control. Scatter graph of topoisomerase II*α* levels in peripheral lymphocytes for all 10 patients at baseline, after topotecan and at the end of treatment (**B**).

The topoisomerase II*α* levels were not increased in peripheral lymphocytes after topotecan treatment (Figure 5). The topoisomerase II*α* levels in 10 patients showed a mean reduction of 2.14 arbitrary units (*P*=0.02) following topotecan, compared to baseline measurements. However, there was no trend for change overall with treatment for all patients or for the subset of those who responded to treatment.

## DISCUSSION

One of the goals of combination chemotherapy is the development of regimens with synergistic activity and noncrossover toxicity. This paper explores the combination of topotecan and EP; from preclinical studies to suggest the most active sequencing of the drugs, through to a phase I study of the combination in patients with advanced ovarian cancer.

Release of tortional stress in supercoiled DNA is necessary prior to replication and cell division. Topoisomerase I induces a single-strand break in DNA, allowing it to unwind, before repair of the break. Topotecan binds and stabilises the topoisomerase I–DNA complex, preventing resealing of the DNA (Hsiang *et al*, 1985). This leads to double-strand breaks, apoptosis and cell death.

Type II topoisomerases make transient breaks in double-stranded DNA (dsDNA). Etoposide binds and stabilises an intermediate enzyme–DNA structure inhibiting further enzyme activity (Chen and Liu, 1994; Froelich-Ammon and Osheroff, 1995). When the cell attempts to replicate its DNA, these complexes are disrupted causing double-strand breaks, which, unless repaired, will be lethal to the cell. Thus, etoposide cytotoxicity depends both on the level of topoisomerase II, which is expressed only in dividing cells in the later phases of the cell cycle (Heck *et al*, 1988), and secondly on duration of exposure of cells to the drug.

The *in vitro* studies described here demonstrate the schedule dependence underlying the interaction between topoisomerase I and II inhibitors (SN-38 was used for *in vitro* experiments as it is also a topoisomerase I inhibitor). These data also demonstrate antagonism when the two agents were used simultaneously, where the expected effect (20% apoptosis) based on the activity of each agent used alone was not observed in any cell line. Increased activity, inducing the greatest percentage of apoptotic cells, was observed with schedules in which the two drugs were given sequentially. The most active combination overall was SN-38 for 3 days, followed by 1 day without drug treatment, and then etoposide for a further 3 days. Cell cycle data (not shown) suggested that the increased apoptotic effect observed with consecutive exposures may be due to relief of the cell cycle block occurring after exposure to the first agent, when that agent is removed and the cells are then exposed to the second agent. This is more marked when the drug-free interval is short. These results led to the adoption of a similar schedule for the clinical trial.

These results are in keeping with previous *in vitro* studies demonstrating synergy when topotecan is followed by etoposide (Bonner and Kozelsky, 1996; Grabowski and Ganapathi, 1996; Chen *et al*, 2002). Synergy has also been demonstrated in human xenograft mouse tumour models (Kim *et al*, 1992; Whitacre *et al*, 1997). Additive effects have also been observed when topoisomerase I and II inhibitors were given sequentially as opposed to simultaneously (Bertrand *et al*, 1992). By contrast, simultaneous incubation of cells with camptothecin (a topoisomerase I inhibitor) and etoposide has shown reduced cytotoxicity compared to etoposide given alone (Kaufmann, 1991).

The preclinical data presented here suggested synergistic activity when cells are treated sequentially with topoisomerase I then II inhibition. The topoisomerase ‘shutdown’ model predicts that treatment of cells with a topoisomerase I inhibitor results in upregulation of topoisomerase II levels, as an alternative DNA repair mechanism is activated. Sequential treatment with a topoisomerase II inhibitor, where the activity is in part dependent on topoisomerase II levels is therefore predicted to be synergistic. However, in a study in patients with advanced non-SCLC, of topotecan 0.85 mg m^−2^ day^−1^ as a continuous i.v. infusion for 24 h followed by oral etoposide 100 mg b.i.d. on days 7–9, only one patient responded to treatment and the authors concluded that in future studies etoposide should be given more closely following the topotecan (Dowlati *et al*, 2001). Etoposide phosphate infusions allow prolonged scheduling of etoposide with pharmacokinetic monitoring to allow dose adjustments. Trials in our unit over the past decade have shown this to be feasible and well tolerated (O'Byrne *et al*, 1997; Joel *et al*, 1998; Braybrooke *et al*, 2003). Experience from previous studies, together with the preclinical data presented here, led to the adoption of the sequential administration of topotecan for 5 days followed by EP for 5 days as a novel regimen to be investigated in this phase I study in patients with advanced ovarian cancer. As anticipated, the major toxicity of topotecan followed by EP was myelosuppression. The MTD was topotecan 0.85 mg m^−2^ day^−1^ followed immediately by a 5-day infusion of EP at a plasma concentration of 1 *μ*g ml^−1^. However, seven out of 11 patients on this dose level completed six cycles, in keeping with this dose level being a feasible dose for further study in a phase II trial. Two commonly used second-line treatments for ovarian cancer are single agent topotecan 1.5 mg m^−2^ day^−1^ days 1–5 q21 and liposomal doxorubicin 50 mg m^−2^ q28. These schedules have been compared head to head in a phase III study (Gordon *et al*, 2001). Table 7 compares the toxicities observed in the Gordon study with those from this study at the dose level suggested for phase II evaluation. The haematological toxicities from this study and the single agent topotecan arm are similar, especially given the use of stem cell growth factor support in the Gordon study. This strengthens the case further for the use of topotecan/EP as reasonable for a palliative regimen.

The combination of topotecan followed by etoposide has been evaluated in other studies. In patients with AML (acute myeloid leukaemia), an MTD of topotecan 1.5 mg m^−2^ day^−1^ continuous infusion days 1–5 followed by etoposide 100 mg m^−2^ day^−1^ × 3 was identified (Crump *et al*, 1999). Mucositis was the DLT with grade 4 neutropenia and thrombocytopenia also observed. Other disease-specific phase I studies of sequential topotecan and etoposide have also found myelosuppression to be dose limiting (Cooper *et al*, 1996; Herben *et al*, 1997; Hammond *et al*, 1998; Crump *et al*, 2002). However, a phase I study in SCLC found topotecan 0.75 mg m^−2^ day^−1^ followed by a 1-h infusion of etoposide 60 mg m^−2^ day^−1^ daily for 5 days in a three weekly cycle was well tolerated (O'Neill *et al*, 2001). Two studies have examined the effect of order of sequential topotecan and etoposide in patients with lung cancer. Patients received the two drugs in the opposite order in subsequent cycles. No difference in toxicity was observed between the different sequences and in both studies, myelotoxicity was dose limiting (Huisman *et al*, 2001; Mok *et al*, 2002).

The response rate in this study was 28% in evaluable patients, of whom half were resistant to platinum. This compares favourably with the response rate of 20.5% for topotecan given second line in advanced ovarian cancer (ten Bokkel Huinink *et al*, 1997). All responding patients had been sensitive to platinum first line. Five of the six responding patients had also been treated previously with paclitaxel, supporting the data that the combination of topotecan and etoposide is noncrossresistant with paclitaxel. Of 18 patients, 12 were evaluable by Ca125 and had a decrease or stabilisation of their levels. Although this has not been shown to correlate with activity, it may be that more minor improvements in disease parameters are clinically relevant in the setting of advanced disease, where prolonged stabilisation is a valid clinical objective. The QOL data showed a trend to improvement during the course of the study. Quality of life scores were statistically related to haemoglobin concentration, as previously described (Littlewood *et al*, 2001).

There are two human type II topoisomerases, *α* and *β*. Topoisomerase II*α* levels vary with the cell cycle (unlike topoisomerase II*β*) and are not detectable in S-phase but increase through the cell cycle, peaking at G_2_/M (Woessner *et al*, 1991). An *in vitro* study demonstrated that topoisomerase II*α* levels could be induced in colon cancer cell xenografts that had been exposed to topotecan. However, if topotecan treatment was withdrawn for 5 days, the topoisomerase II*α* level fell back to baseline (Whitacre *et al*, 1997). This further supports that etoposide should administered immediately follow topotecan, as in this study.

Topoisomerase II*α* levels measured during this study did not show any significant changes from baseline to the end of treatment with topoisomerase I then II inhibition, although there was a trend for topoisomerase II*α* levels to fall following topotecan (Figure 5B). However, the topoisomerase II*α* were measured in circulating lymphocytes, which are not undergoing cell division. Only cycling cells are expected to have high levels of topoisomerase II*α* and to be sensitive to topotecan and this may account for our findings in peripheral blood.

Topoisomerase II*α* levels measured in the tumour cell lines did rise following SN-38 and fell again following etoposide, as predicted in the topoisomerase ‘shutdown’ model (data not shown). Similar results have been demonstrated in CML K562 cells treated with topoisomerase I then II inhibitors (Chen *et al*, 2002). A rise in topoisomerase II*α* has also been observed in peripheral blast cells from leukaemia patients by day 3 of treatment with topotecan, although levels returned to baseline by day 5 (Crump *et al*, 1999). In another study in leukaemia patients treated with topotecan then etoposide and mitoxantrone, a rise in topoisomerase II*α* post topotecan correlated with response to treatment (Mainwaring *et al*, 2002). A further study using fluorescence cytometry to measure nuclear topoisomerase II*α* levels in cells from patients with AML also showed a rise in the median value after 48 h of treatment with topotecan, falling again by 5 days, although marked interpatient variability was observed (Nicklee *et al*, 1996). Topoisomerase II*α* levels have also been measured in tumour biopsy specimens, pre and post topotecan and etoposide treatment, in four patients as part of a phase I study. There was no convincing pattern of change, in particular three out of four patients' levels were unchanged following topotecan administration; however, these numbers are very small (Hammond *et al*, 1998).

Separately, topotecan at 1.5 mg m^−2^ day^−1^ and EP at a plasma etoposide concentration of 3 *μ*g ml^−1^, each over 5 days, can be given with very manageable toxicity. In this study, substantially lower doses of both topotecan and etoposide given sequentially resulted in greater than anticipated haematological toxicity. Furthermore, there was a higher tumour response rate (28% in evaluable patients, 24% if all 21 patients were included) than would be expected for the combination of drugs at these subtherapeutic doses. Together with the preclinical data, the toxicity and response rate from this study argue for synergy of action between these drugs.

A shorter course of topotecan 1.5 mg m^−2^ day^−1^ for 3 days, q21 in platinum and paclitaxel-refractory ovarian cancer has been investigated (Markman *et al*, 2000) and the 3-day topotecan programme was found to be more convenient and less toxic than the standard 5-day regimen with apparently comparable activity. From this study, the recommended dose for a phase II trial is topotecan 0.85 mg m^−2^ day^−1^ for 5 days followed immediately by a 5-day infusion of EP at a plasma concentration of 1 *μ*g ml^−1^. However, in view of the considerable haematological toxicity in this study and the results from Markman's study (Markman *et al*, 2000), a 3-day topotecan 0.85 mg m^−2^ day^−1^ schedule followed by a 5-day infusion of EP at 1 *μ*g ml^−1^ might be preferable. In future studies, it would be of particular interest to measure topoisomerase II*α* levels in cycling cells, either from tumour biopsy specimens or in cells collected from patients with ascites.

## Figures and Tables

**Table 1:** Topotecan and etoposide dose levels

**Dose level**	**Topotecan (mg m^−2^ day^−1^)**	**Etoposide level (*μ*g ml^−1^)**
1a	1.0	2.0
1b	0.85	2.0
2a	0.85	1.0
2b	0.85	1.5

**Table 2:** Patient details

**Age and PS**		**Histology**		**Stage at presentation**		**Previous surgery**	
Number of patients, *n*	21	Ovary – serous	12	Stage I	0	TAH, BSO and omentectomy	9
Age – mean	54	Ovary – endometroid	4	Stage II	1	BSO and TAH	3
Age – range	31–66	Ovary – clear cell	1	Stage III	14	BSO	4
PS=0	6	Ovary – mucinous	1	Stage IV	6	BSO and omentectomy	2
PS=1	11	Primary peritoneal – serous	1			RSO and omentectomy	1
PS=2	3	Primary peritoneal – clear cell	1			Biopsy only	2
PS=3	1	Fallopian tube – endometroid	1				

TAH=total abdominal hysterectomy; BSO=bilateral salpingo-oophorectormy.

**Table 3a:** Previous treatment – first-line chemotherapy

	**Carboplatin**	**Carboplatin+** **paclitaxel**	**Cisplatin-** **containing** **regimens**
*Number of patients*	12	7	2
CR	5	4	2
PR	3	3	0
SD	2	0	0
PD	2	0	0
Platinum sensitive	7/12	3/7	1/2
Mean duration of first remission (min) (excluding patients with PD)	18.0	6.3	9.0

CR=complete remission; PR=partial remission; SD=stable disease; PD=progressive disease.

**Table 3b:** Previous treatment – second- and third-line chemotherapy

	**Second-line** **chemotherapy**					**Third-line chemotherapy**				
**Treatment**	**Total**	**CR**	**PR**	**SD**	**PD**	**Total**	**CR**	**PR**	**SD**	**PD**
Carboplatin	3	1		1	1					
Paclitaxel	4		2	1	1	2	1	1		
Carboplatin+paclitaxel	2		2							
CAP	2				2					
FEC	1				1					
Cyclophosphamide+epirubicin	1				1					
Oral etoposide						1			1	
Noncytotoxic – trial treatment	2	1		1		1	1			

CR=complete remission; PR=partial remission; SD=stable disease; PD=progressive disease.

**Table 4:** Toxicity data

**Dose-limiting toxicity (following cycle 1 only)**					
**Dose level**	**Number of patients (*n*)**	**Thrombocytopenia**	**Neutropenia**	**Sepsis**	
1a (1.0/2.0)	2	1	2	2	
1b (0.85/2.0)	3	1	3	3	
2a (0.85/1.0)	11	0	4	1	
2b (0.85/1.5)	5	4	4	4	
					

**Haematological toxicity over 6 cycles**					
**Dose level**	***n***	**Anaemia**	**Thrombocytopenia**	**Neutropenia**	**Sepsis**
1a (1.0/2.0)	2	0	1	2	2
1b (0.85/2.0)	3	0	2	3	3
2a (0.85/1.0)	11	2	3	6	5
2b (0.85/1.5)	5	0	4	5	4
					

**Nonhaematological toxicity over 6 cycles**					
**Dose level**	***n***	**Alopecia grade ⩽2**	**Nausea grade=3**	**Stomatitis grades 2–3**	**Diarrhoea grades 2–3**
1a (1.0/2.0)	2	2	0	2	1
1b (0.85/2.0)	3	2	1	2	1
2a (0.85/1.0)	11	11	2	2	0
2b (0.85/1.5)	5	5	0	1	0

Only grade 4 haematological toxicity presented.

**Table 5:** Patients requiring dose adjustments of etoposide phosphate following measured etoposide levels more or less than 10% from the target level

**Cycle** **number**	**No. of** **patients** **treated**	**No. of** **patients for** **whom data** **available**	**Patients** **requiring dose** **adjustment on** **day 2**	**Patients** **requiring dose** **adjustment on** **day 4**
1	21	21	18	4
2	21	19	15	8
3	18	18	12	8
4	14	13	6	6
5	14	14	12	4
6	12	12	7	7

**Table 6:** Response data for evaluable patients (⩾3 cycles of treatment)

		**Response to treatment**				
**Dose level**	***n***	**PR**	**SD**	**PD**	**Platinum** **resistant**	**Previous** **paclitaxel**
1a (1.0/2.0)	2	2	0	0	0	2
1b (0.85/2.0)	3	0	1	2	2	3
2a (0.85/1.0)	9	2	3	4	4	5
2b (0.85/1/5)	4	1	2	1	1	4
Total	18	5	6	7	7	14

PR=partial remission; SD=stable disease; PD=progressive disease.

**Table 7:** Comparison of percentage of patients with grade 3/4 toxicities with that of Gordon *et al* (2001)

	**Topotecan** **(0.85 mg m^2^ day^−1^** **days 1–5, EP** **(1 *μ*g ml^−1^) days** **6–10**	**Topotecan** **(1.5 mg m^2^ day^−1^)** **days 1–5 q21**	**Liposomal** **doxorubicin** **(50 mg m^−2^)** **q28**
Neutropenia^a^	82	77	12
Anaemia	45	28	5
Thrombocytopenia	45	34	1
Plantar/palmer erythema	0	0	23
Stomatitis	0	0.4	8

a Growth factor support was used in the study of Gordon *et al* (2001).
